# Treatment “non-responders”: the experience of short-term psychoanalytic psychotherapy among depressed adolescents, their parents and therapists

**DOI:** 10.3389/fpsyg.2024.1389833

**Published:** 2024-09-19

**Authors:** Guilherme Fiorini, Zane Khoe, Peter Fonagy, Nick Midgley

**Affiliations:** ^1^Division of Psychology and Language Sciences, Research Department of Clinical, Educational and Health Psychology, University College London, London, United Kingdom; ^2^Anna Freud, London, United Kingdom

**Keywords:** psychoanalytic psychotherapy, adolescents, depression, qualitative methods, multiple informants

## Abstract

**Introduction:**

Short-term psychoanalytic psychotherapy (STPP) is an evidence-based treatment for adolescents with depression, but like all treatment approaches, not all patients benefit from it. Previous investigations of the process of STPP have mostly focused on successful cases, and only a few studies have included the perspectives of young people, their parents, and therapists in the understanding of treatment non-response.

**Methods:**

Semi-structured interviews were carried out with young people who were considered “non-responders” to STPP, as well as with their parents and therapists. These cases were analyzed using a descriptive-interpretative approach.

**Results:**

The data analysis revealed three themes: (1) Therapy as a safe space; (2) Can short-term psychotherapy ever be enough?; and (3) Therapists making links and connections that did not make sense to the young people.

**Discussion:**

This study’s findings indicate that “poor outcome” psychotherapy does not necessarily equate to a “poor experience” of psychotherapy, with different stakeholders appreciating the treatment setting as a “safe space.” However, they also suggest that some felt that a relatively short-term treatment could not lead to substantial change and that young people in STPP might have a more negative view of their outcomes compared to their parents and therapists. Finally, the findings indicate that some interventions made by clinicians in STPP feel wrong or do not make sense to young people, potentially affecting the therapy process.

## 1 Introduction

Up to a third of clinically depressed adolescents who go through psychoanalytic psychotherapy end up not showing any indications of improvement in depressive symptoms (National Institute for Health and Care Excellence, 2019; Midgley et al., 2021), as also observed in several alternative treatment approaches (Cuijpers et al., 2020). In this context, whilst previous literature has mostly focused on understanding what are the characteristics of *successful* treatments, fewer studies have paid attention to examining the interventions that *do not* work (Barlow, 2010). Understanding what is associated with unsuccessful therapies might be key to informing clinicians and researchers about what features may hinder patient response, leading to improved treatments, or at least drawing more parsimonious goals and adaptations in current practices.

Prior investigations have evidenced some predictors that are associated with poor outcomes in adolescent psychotherapy. For example, young people with higher levels of psychological impairments seem to be less likely to improve after receiving a range of mental health treatments when compared with less impaired youth (see Cervin et al., 2021; Edbrooke-Childs et al., 2022; Fiorini et al., 2023b). Likewise, patients with lower motivation to change or engage in therapy tend to achieve poorer outcomes (Fitzpatrick and Irannejad, 2008; Black and Chung, 2014). Nevertheless, these baseline indicators only throw light on the response likelihood for a given patient in comparison to broader populations and do not capture some relevant variables involved in therapy (Midgley et al., 2017).

Besides the patients’ presentation at baseline, some studies have indicated that features that take place during the therapy process could also influence patient response. The literature on the therapeutic alliance, for instance, has demonstrated that adolescent-therapist alliance is associated with outcomes (McLeod, 2011; Shirk et al., 2011; Karver et al., 2018), even though this seems to work differently depending on the therapy modality being used (Cirasola et al., 2021).

Specifically concerning features associated with “unsuccessful” psychoanalytic psychotherapy with adolescents, the study performed by Fiorini et al. (2023a) has indicated that young people who express higher levels of in-session anger seem to achieve worse outcomes. While this finding might contradict the idea that psychoanalytic psychotherapy should help adolescents being able to express their anger (Cregeen et al., 2017), the authors suggest that what “is done” with this expressed anger might also be important. Perhaps these angry feelings were too overwhelming and processing them was too challenging for the young people. Additionally, clinicians could have struggled to address these feelings in a therapeutic way (see Chourdaki et al., 2023).

Despite the relevance of these investigations for our understanding of “successful” and “unsuccessful” psychotherapy, they often rely on self-report questionnaires and the perspectives of external examiners. This framework leads to a limited understanding of the multiple and complex phenomena involved in psychotherapy. In that sense, qualitative investigations, including stakeholders’ own perspectives on a lived experience could shed light on treatment aspects that may be overlooked by other methods.

Concerning young people’s subjective perspective on psychoanalytic psychotherapy, a meta-synthesis reported by Fiorini et al. (2024) has gathered some initial insights. Firstly, adolescents seem to appreciate different facets of the therapy relationship. That included perceiving the therapist as someone who is warm, caring, and who would be available to “hear” them. This study also evidenced that many patients perceive psychoanalytic psychotherapy as a painful process, in which they have to access troublesome feelings and expose themselves. Lastly, this review has also indicated that some young people feel like they need to “navigate” their role as patients in these treatments, including making sense of how psychotherapy should unfold and how they should behave in the setting. Although this review points to relevant aspects of adolescents’ experience of therapy, a few aspects should be highlighted: (1) overall, most of the studies included did not address the treatments’ outcomes, so little is known about how these perceptions on the relationship, the experience of therapy being painful, and the process of “navigating” one’s role in psychotherapy relate to outcomes; (2) only one of the studies included (i.e., Housby et al., 2021) explicitly focused on good outcome cases, but it is unclear how the experiences of “successful” cases would relate to the experience of “unsuccessful” ones; (3) no studies focused on poor outcome cases.

In one of the few studies employing qualitative methods to understand poor outcome psychotherapy cases in the treatment of adolescents, Mehta et al. (2023) analyzed interviews with five young people who participated in the IMPACT trial. Their main findings indicated that these young people considered their depression too overwhelming for them to be “cured” by what therapy can offer. They also reported that therapy could make them feel worse, including feeling like a burden or having a negative experience regarding the therapy relationship. Finally, the authors also found that despite being classified as “non-responders” by standardized measures, some adolescents would refer to some small improvements such as having better self-awareness or feeling allowed to share their thoughts and feelings (Mehta et al., 2023). These findings provide valuable insights for the understanding of treatment “failure.” Nonetheless, they include the perspectives of young people attending different treatments (i.e., short-term psychoanalytic psychotherapy—STPP-, cognitive-behavioral therapy-CBT-, and a brief psychosocial intervention-BPI), with only one going through STPP. Therefore, we do not know if these experiences are modality-specific or more generalized among “poor outcome” cases.

Alongside the relevance of giving voice to young people’s perspectives on their treatments, it is also important to consider that psychotherapy is a process that implicates different stakeholders in its nature. In a study performed by Werbart et al. (2019), it was in fact evidenced that addressing the intersection of different perspectives can also be crucial to foster a better understanding of therapy “success” and “failure.” In this investigation, the authors analyzed interviews with 3 psychoanalytically oriented therapists, alongside two patients for each one of them (one being a “good outcome” case and the other a “poor outcome” one, making up to six patients in total). The authors’ analysis suggested that therapists and patients in “successful” cases would share a more congruent understanding of the presenting problems and the treatment goals. Also, in the “good” outcome cases, the dyad would experience their relationship and the psychotherapy process as supportive and challenging, and the therapist would adapt their technique according to the patient’s needs. Conversely, “poor” outcome cases were characterized by a dissonance between the dyad’s understanding of the process and outcomes. Therapists were more prone to attribute the difficulties in the process to the patient and less prone to adapt their technique, and to consider their own role in the therapy “failure” (Werbart et al., 2019). Despite these important contributions, it is unclear how these perspectives would be found in the context of psychotherapy with young people. This is particularly relevant because adolescence has specific developmental challenges that might impact the psychotherapy process. By usually being a life period of separation between the young person and their primary adult figures (Jager et al., 2015), these treatments might entail a perceived power imbalance between the young client and their (adult) therapist (Fiorini et al., 2024) that could in turn affect outcomes.

Besides the relevance of young people and therapists and their perspectives concerning psychotherapy, it is worth noting that parents are also key actors in these treatments. Firstly, parents usually have substantial involvement in the therapy process and can play a role in treatment continuation: besides being a usual source of referral, they may be the ones paying for the treatments, and providing transportation (Hawley and Weisz, 2005). Secondly, according to a meta-analysis performed by Karver et al. (2018), the alliance established between parents and therapists is as important as the alliance between children and therapists in terms of their relationship with outcomes. In that sense, parents are crucial actors that should be included in research addressing youth psychotherapy.

Considering the factors concerning young people, therapists, and parents and their association with outcomes, one can infer that treatment effectiveness can be affected by multiple factors. Furthermore, the literature points out that any one perspective is likely to provide only a partial understanding if looked at in isolation. Therefore, studying the viewpoint of different stakeholders involved in a given treatment could be key in providing a more rounded understanding of the interventions provided (De Los Reyes et al., 2015). Additionally, many studies exploring psychotherapy failure have relied on standardized measures, including patient self-report questionnaires or observer-rated assessment tools. Although standardized measures are useful in mapping general aspects of psychotherapy, they do not provide the full picture of the patients’ sufferings (Krause et al., 2019, 2020), with qualitative methods being potentially useful in achieving a more meaningful understanding of what kind of outcomes matter most to patients (McLeod, 2013). Considering this background, the present study aimed to understand the experience of short-term STPP for depressed adolescents who remained clinically depressed after therapy ended, including the subjective perspectives of patients, parents, and therapists. Given the qualitative and exploratory nature of this study, we addressed the aim according to the following research question: “how do young people who did not respond to STPP, their parents, and therapists make sense of their experience of psychotherapy?”

## 2 Methods

### 2.1 Design

This study was drawn from a larger investigation, namely the IMPACT-My Experience (IMPACT-ME; Midgley et al., 2014) study. The IMPACT-ME study was a qualitative investigation embedded in a larger trial, the Improving Mood with Psychoanalytic and Cognitive Therapies (IMPACT) study (Goodyer et al., 2017), assessing the treatment and relapse prevention of depression in young people. Within the IMPACT-ME study, young people, their therapists, and their parents were interviewed at three different time points, following semi-structured protocols (see more details in the “Data collection” section). In this particular investigation, we focused on examining the experience of STPP of young people who remained clinically depressed after therapy ended, their parents, and therapists. For more information about the IMPACT-ME study see Midgley et al. (2014) and Goodyer et al. (2017).

### 2.2 Participants

The participants included in this study are a sub-sample of a previous investigation that compared the in-session interactions in “successful” and “unsuccessful” psychoanalytic psychotherapies (Fiorini et al., 2023a). In this previous study, after applying data availability criteria (including randomization status, location, and number of session recordings), 22 young people were identified. Out of these 22 adolescents (including cases of “good” and “poor” outcomes), the 5 with the highest likelihood of experiencing a “poor outcome” trajectory of change in terms of their general psychopathology (or *p* factor, calculated via a latent class growth analysis) were chosen. Since the *p* factor scores were an aggregation of different symptoms available in the dataset (in this study: depression, anxiety, obsessions and compulsions, and conduct problems), these young people were the most likely to have an overall poor trajectory when taking into consideration all domains. For a more thorough description of the selection criteria see Fiorini et al. (2023a), and for more information on the latent class growth analysis see Fiorini et al. (2023b).

Out of the five “poor outcome” cases described in Fiorini et al. (2023a), one was excluded from the present study for not having IMPACT-ME interviews available. The four selected cases described in this investigation encompassed a sub-sample of adolescents from the IMPACT/IMPACT-ME studies who presented clinical levels of depression before and 1 year after attending STPP, and their respective parents and therapists. All participants completed their treatment (i.e., they did not drop out), despite this not being an inclusion criterion. The young people’s demographic characteristics and depressive symptom ratings are presented in Table 1 (all names are pseudonyms). In this study, each case had a different psychotherapist (i.e., no psychotherapist within this study treated more than one young person).

**Table 1:** Young people characteristics.

Name	Daniel	Riley	Marcus	Anna
Baseline age	16	16	14	17
Ethnicity	White British	White British	Mixed	White (other)
MFQ baseline	29	61	51	56
MFQ Week 6	20	n/a	45	n/a
MFQ Week 12	43	41	37	39
MFQ Week 36	41	48	38	40
MFQ Week 52	46	45	49	25
MFQ Week 86	29	n/a	36	43

MFQ, Mood and Feelings Questionnaire (scores above 27 are considered within the clinical range).

All young people selected presented clinical depression levels before therapy and in their respective last assessment, as measured by the Mood and Feelings Questionnaire (MFQ; Wood et al., 1995). Although three of them did show some reduction in their MFQ scores by the one-year follow-up, compared to baseline, and none showed deterioration in their depressive symptoms from baseline to 86-week follow-up, they all met criteria for belonging to the “unsuccessful” outcome group as measured through a latent class analysis published elsewhere (Fiorini et al., 2023b). Furthermore, the symptom trajectory presented in Table 1 illustrates that these young people’s depressive symptom scores oscillated over time points.

### 2.3 Intervention

All young people included in this study were randomized into short-term psychoanalytic psychotherapy (STPP; Cregeen et al., 2017). STPP is an intervention aimed at helping the patient to give meaning to their emotional experiences, attachment patterns, and developmental tasks. STPP includes reflections on the therapeutic relationship and uses supportive and expressive strategies to help the young person. Therapists in this modality should keep a non-judgmental and enquiring stance (also called a “psychoanalytic stance”), trying to convey through words what the adolescent is communicating consciously and unconsciously. STPP included up to 28 weekly individual sessions plus seven parent/guardian sessions offered by a different clinician. All STPP therapists were Child and Adolescent Psychotherapists working in the National Health Service (NHS) centers who were part of the study.

### 2.4 Data collection

The interviews examined in this study took place between the years 2011 and 2014. For each case, they were held at two different time points: either right after the end of therapy (T2) and at a one-year follow-up (T3). The interviewers were all post-graduate psychologists working on the IMPACT-ME study. They followed a series of semi-structured interview schedules, having received a half-day training session for conducting them.

For T2, the interview schedules were named *Experience of Therapy Interview*, and they were carried out separately with young people, parents, and (where the young person gave permission) therapists. They addressed the participants’ perspectives on (a) what were the difficulties of the young person that led them to seek a Child and Adolescent Mental Health Services (CAMHS); (b) how they understood these difficulties; (c) any perceived changes within the last calendar year; (d) the “story” of therapy, including the participants’ impressions on the therapy relationship, and any subjectively meaningful moments; their evaluation of psychotherapy including their understanding if therapy was helpful or unhelpful, and in what aspects; (f) their experience of involvement in taking part in a clinical trial.

The *Thinking back about therapy interview* (T3) schedule was used with YP and parents, and most of its items were a review of the ones addressed in T2. It encompassed the participants’ perception of (a) how was life since the last interview; (b) their current understanding on what were the difficulties that led the young person to seek help from CAMHS; (c) “thinking back about therapy,” focusing on the participants’ recollection about the experience of therapy; (d) any links between therapy and change/no-change; and their experience of taking part in a clinical trial.

The interviews took place at a CAMHS of choice of the participants or their residence, and took, on average, 1 h each (range: 30–103 min, *M* = 69.15 min). They were audio-recorded and transcribed verbatim, hiding any identifying information such as names, or places. Young people were invited to choose a pseudonym for themselves to be used in any publications The interview availability per case is presented in Table 2.

**Table 2:** Interviews availability per case.

Case name	Young person		Therapist	Parent	
	T2	T3	T2	T2	T3
Daniel	X	X	X	X	
Riley	X				
Marcus	X	X	X	X	X
Anna	X	X	X		

### 2.5 Data analysis

The data analysis followed a generic descriptive-interpretative approach (Elliott and Timulak, 2005; Timulak and Elliott, 2019). This was chosen given the considerable overlap between different qualitative analysis “brand names” (e.g., grounded theory, interpretative phenomenological analysis, thematic analysis, among others), which involve describing and interpreting a phenomenon of interest (Timulak and Elliott, 2019).

This analysis has a focus on understanding individuals’ lived experience, and in the psychotherapy field that is usually applied to patients and therapists. However, while in clinical settings it is widely recognized the parents’ and carers’ role in the psychotherapy process, their perspectives are often overlooked in qualitative studies. With that in mind, this study employed a multiple perspectives design.

In this investigation, the analysis followed several steps. Firstly, the interviews were separated and organized into “clusters,” comprised of the different data points from the same case (i.e., since this study reports on four cases, we had four “clusters.” Each cluster included all information available from the same case, such as the interviews with a young person, their parent(s), and/or their respective therapist). After organizing each cluster, they were analyzed in a standard order. In this process, two researchers (GF and ZK) started by independently reading and listening to the interviews of one young person (that is T2 and/or T3 interviews with a singular young person, analyzed jointly when both were available). During and after listening to and reading the interviews, they made independent annotations that were exported to Microsoft Excel and then tabulated tentative themes from them. After organizing the tentative themes for a young person within a cluster, the researchers discussed the themes with each other reaching a shared understanding or consensus. The same process was then repeated for the interviews regarding the same cluster’s therapists and parents. Once all interviews for a single case/cluster were analyzed (including tabulating and organizing tentative themes on Microsoft Excel), the main themes for each “cluster” (i.e., overarching themes drawn from the interviews with the young person, therapist, and parent from a single case, combined) were delineated. This process was repeated for all subsequent cases/clusters. After delineating each cluster-level themes, they were examined jointly and organized in a general matrix, tabulated in Excel. The themes comprising the general matrix were examined in terms of how they represented each case, and each “grouped” perspective (e.g., how each theme was understood by different participants, such as young people, therapists, and parents), and then described in the results section. In different stages of the analysis, NM (an expert in qualitative methods) audited the themes, in order to ensure their precision, clarity, and their alignment with this study’s research aim.

### 2.6 Ethical procedures

The IMPACT-ME study protocol was approved by the Cambridgeshire 2 Research Ethics Committee, Addenbrookes Hospital Cambridge, UK (REC Ref: 09/H0308/137). All participants have provided written consent to participate in the study. Aiming to ensure confidentiality, identifiable details were excluded or concealed in this report.

## 3 Results

Following the data analysis and considering our exploratory research question, we formulated 3 main themes across cases, each describing one facet of the experience of STPP for the participants. The themes are:

1. Therapy as a safe space.
2. Can short-term psychotherapy ever be enough?
3. Therapists making links and connections that did not make sense to the young people.

The themes encompassed some experiences that are consistent throughout the perspectives of young people, therapists, and parents, while others described perspectives that are contrasting between participants or specific to a determined group, as detailed below.

### 3.1 Theme 1: therapy as a safe space

Theme 1, named “Therapy as a safe space” was the most commonly and consistently described among participants, being found in all interviews. In this theme, participants depicted and appreciated therapy as a place where the young person would express themselves or talk about subjects that would not be possible in other contexts. Furthermore, as explained by Daniel, therapy was a place where they did not feel judged, perhaps even allowing for the reflection on their own behaviors:

**Daniel:** It’s nice to tell someone who’s not gonna be like ‘oh you shouldn’t have done that’ or ‘that was really stupid of you’ … cos there are a lot of things I’ve done that was really dumb, shouldn’t have done that, that was really stupid. Whereas when I tell her, she goes ‘and what do you think about that?’ and I go ‘it was really fucking stupid’ but it’s better than her like going to me ‘that was stupid’ cos if I say it, it makes me feel better instead of someone telling me I was stupid.

In addition, this sense of a safe space was also understood as including a therapist stance of respecting the young person’s time and readiness to discuss certain topics:

**Riley:** She’ll know … if I don’t wanna talk, she won’t push me, she’s happy just to sit there but I think she can tell when I’m more open to discussing things and when I just wanna be left alone, so it depends.

Besides agreeing with young people and describing therapy as a safe space, parents also pointed out the differences between their parenting roles and the therapists’ roles:

**Marcus’s Mother:** He said, ‘it’s good to have someone to talk to from time to time …’, that’s what he said … and … I don’t take it personally, ‘cause I know what he means, there are some things you don’t wanna talk to your parents about … and I think he obviously feels that it’s a safe space for him to talk …

Whilst Marcus’ mother raised her son’s possible internal motivations for not wanting to talk about some subjects with her, Daniel’s mother also highlighted some external boundaries that limit what and how the young person can express outside therapy:

**Daniel’s Mother:** I know [therapy] is a forum where he feels he can … go into the therapist’s room and express … if he’s angry, she [the therapist] allows him to swear and shout and all those kinds of things. Whereas in the family home … it’s not so free for him to be … shouting and swearing.

Furthermore, in some cases the therapy setting was seen as a place for emotional discharge, where one could let out feelings that could be overwhelming:

**Daniel:** I usually [left the sessions] in a good mood. I don’t feel very good during it but feel in a much better mood afterwards … cos I say all the things that make me feel upset there, and then I come out and then I’ve said everything so I kind of feel better.

In this example, Daniel described session dynamics where he would use therapy as a space to unleash his upsetting feelings, promoting some sort of emotional relief by the end of the sessions. Similarly, Anna’s therapist also described comparable interactions, while also highlighting some changes in this over time:

**Anna’s therapist:** I think that she did come to see me as … a sort of touchstone in the week … She could just come and … collapse, really. Cos she did drive herself very hard, y’know in terms of, work and energy, and often she looked absolutely exhausted. And she would … just come and collapse, and for the first part of the treatment it was, usually… great distress and tears. And towards the end, it was much more, kind of … relief.

Lastly, the experience of therapy as a safe space was also fostered by providing some clear boundaries in the therapy setting, according to some participants’ comments:

**Riley:** She knew to ask me like straightforward questions rather than ones that could have any answer. She knew that I liked to have like simple, like to the point questions rather than … people like mixing their words and making it ambiguous.

This young person reported valuing her therapist’s attitude of attuning to her necessities, asking clear and delineated questions, in opposition to open or ambiguous ones. For her, perhaps a therapy setting that presented itself as too open could be felt as too menacing or threatening. Similar remarks could also be found in the perspective of therapists: Daniel’s therapist reports becoming more active in the therapy setting, depending on the patient’s presentation:

**Daniel’s therapist:** There were times when he was too depressed to really talk, and he would often then sort of sit with his head down on his knees and I would have to do quite a lot of the sort of talking for him. But he was quite responsive and … he could describe quite a bit what he was experiencing.

In sum, all these examples illustrate how the different stakeholders understood therapy as a safe space, considering a range of qualities that made them experience it as such. According to the participants, this setting was experienced as a place where the young people could express the thoughts and feelings they considered important or necessary in their own time. Psychotherapy also felt like a place where some young people could “let out” their feelings, especially negative ones. Lastly, some therapists would shift from a more traditionally “open” psychoanalytic stance to a more direct one, aiming to provide clearer direction in the setting, whenever they felt it would be helpful for their patients.

### 3.2 Theme 2: can short-term psychotherapy ever be enough?

In the second theme, named “Can short-term psychotherapy ever be enough?”, the participants provided their own understanding of the treatment’s potential to help the young person overcome depression. This theme was broadly characterized by a dissonance between the young people’s interviews, who described a degree of fatalism or understanding of certain limitations regarding their treatments, and the therapists’ and parents’ interviews, who showed a more positive and optimistic stance.

Some young people reported that they did not seem to believe therapy could help them overcome their problems. For example:

**Marcus:** Well, I just … I felt like by doing this I was - it didn’t feel like it would benefit me in any way cos I guess I couldn’t see the benefit so … I couldn’t tell if anything was changing. It just felt like something extra I had to do rather than something I knew would be helping.

From Marcus’ perspective, going to therapy seemed like a part of his routine that did not help solve his difficulties. According to him, any potential changes were not personally perceived. Along similar lines, Riley stated:

**Riley:** I dunno, I just don’t … feel – I don’t see how an hour a week with someone is meant to change things, especially if you’ve been feeling it for such a long time and you see these people for such a short amount of time … I don’t think it has the potential to do anything at all.

According to this young person, therapy was seen as a limited intervention especially when put into perspective with their overall problems. In this case, their depressive symptoms were present for a significant time before therapy started and were part of their daily life for much longer than the weekly therapy hour offered.

Both Marcus and Riley seemed to have experienced STPP with a sense of hopelessness from the onset of their treatments. In their remarks, the magnitude of their issues was not felt to be possible to be tackled with therapy, and this was reported with a sense of impotence—maybe regarding the patients themselves or the treatments’. Even though the same young people appreciated therapy as a safe space, as presented in theme 1, their treatment process was also seen by them as “pointless,” incapable of producing any type of noticeable improvement.

Conversely, Daniel and Anna experienced therapy as a helpful tool. However, this helpful quality had its limitations and was not seen as sustainable over time:

**Daniel:** When I miss therapy I feel shit, I’m not entirely sure why, but I do. So, I want to keep having it until I can deal with things without it. Which I can’t really at the moment.

According to Daniel, on the days he would miss his therapy appointment he would feel worse. This scenario made him feel he was not ready to manage his feelings without therapy when the program offered ended. Anna also reported her own understanding of the limitations this short-term approach had in helping her:

**Anna:** I would say … [therapy] did impact my life, and it’s always gonna be there somewhere, but also … that it’s kind of had … short-term effects on me, and … it’s hard to say because … it could be my fault that I got depressed again like … it’s … always gonna be there and it helped me a lot … but I think it’s my fault that I couldn’t make it last longer … I don’t know use I couldn’t deal with, I kind of lost control maybe again about dealing with my problems.

In Anna’s case, it is worth noting that by the end of therapy, she showed sub-clinical levels of depression, with an MFQ score of 25 (one of the only two sub-clinical scores in this whole group, considering all time points). However, consistently with her own reports, at a one-year follow-up, she showed an increase in her symptom levels, having her highest scores since baseline. Both Anna’s interviews and her depressive scores indicate that she benefited from psychotherapy, but those benefits did not last.

Daniel’s and Anna’s reports depict how these young people managed to experience therapy as beneficial, but only while it lasted or at least not sustained after 1 year. These young people did not seem to be ready to end psychotherapy after the short-term program offered, still in need of a space to let out their negative feelings or reflect on life decisions with someone else in a supportive setting. Furthermore, the young people’s remarks also suggest some degree of guilt concerning their own outcomes: according to their perspectives, it was not therapy that “failed them,” but rather “they failed” to sustain their treatments’ aid.

Contrasting with the young people’s reports, therapists and parents seemed to have a more positive understanding of therapy as a beneficial experience, not focusing on the potential limitations of the treatment approach. Marcus’ therapist, for instance, reported:

**Marcus’ therapist:** I mean in terms of *presentation* he changed quite a lot … in terms of what he was managing to do … like … going to school … writing, taking part in outside things, the things he’d not done at all before … I think … he’d developed a little bit more understanding of what some of this was about … but also a bit more therefore flexibility … in a way that it didn’t have to be … everything or nothing.

In this extract, Marcus’ therapist highlighted positive changes that were observable both from a behavioral level but also from the young person’s internal functioning. According to her, Marcus resumed the activities he used to do before the onset of his depression and seemed to engage in more mature and less fragmented thought processes. Within the same domain, Daniel’s therapist added:

**Daniel’s therapist:** He did manage to … be able to look back at his depression by the time we ended and see how depressed we had been and … he did much better in his educational … achievements than I think he’d thought he could … The story I think was a very good outcome for this particular [young person] because he had insight and he also appreciated he cottoned on to transference in … understanding about what was going on in the relationship with me and who he saw me figuring as in a way which worked very well for him.

In this case, Daniel’s therapist pointed to academic achievement as one indication of improvement. Furthermore, according to her perspective, the young person managed to develop his insight capacity and use the transference work as a learning tool.

Overall, all therapists’ reports included broad criteria for assessing the young people’s improvement: academic success, engagement and re-engagement in activities, flexibility when dealing with personal issues, self-understanding, and reflection on relationships. Along the same lines, parents also described noticing a positive change:

**Marcus’ mother:** Well, he’s certainly … not in that dark place … and what I think is most important … is that he can now say ‘this is upsetting me, that is making me angry’ … he’s actually now able to analyze some of his feelings … for example … he says ‘before I explode or before I get angry I go and take a walk’ and so to me, he’s made a lot of progress … from being depressed but also … analyzing what he’s feeling at the moment.

Marcus’ mother noticed improvements in her son’s capacity to express his own feelings but also considered that his depressive symptoms decreased. Her descriptions of her son’s capacity to “analyze” his emotions seemed to describe Marcus’ increased skills for self-reflection and self-regulation. However, even though she directly attributed the positive change and these skills’ development to psychotherapy, this was not true for all cases:

**Daniel’s mother:** There was a huge amount of positive change. [Interviewer: what would you say were the most important reasons for that change?] I think he thought-it was his perceptions-he thought that… his depression had been caused by his GCSEs … were over.

In this excerpt, Daniel’s mother reported that her son attributed his problems to the stress caused by the preparation for his GCSEs, and the passing of the GCSEs as the reason why the problems diminished. Even though she explicitly considered therapy as necessary in her son’s life during her interview, she did not associate his life changes directly with the treatment process.

### 3.3 Theme 3: therapists making links and connections that did not make sense to the young people

The third and last theme, “Therapists making links and connections that did not make sense to the young people,” was comprised of the young people’s perspectives only, and did not appear as a theme in the parent or therapist interviews. This theme describes the adolescents’ experience of not understanding the reason for some interventions, or appreciating some of them as unhelpful or inappropriate during their therapy process:

**Anna:** I kind of still don’t understand is how she always… tried to see my relationship with other people through my relationship with her…

From this excerpt, we can notice that Anna described not understanding the reason why her therapist would frequently try and establish connections between their relationship and the patients’ relationships outside psychotherapy. According to these young people, not understanding these connections was not the only issue concerning the discussion of the therapy relationship, as they were also sometimes perceived as constantly inaccurate:

**Marcus:** She linked a lot of things to go into therapy … and … sometimes it just didn’t feel like that at all, a lot of the time.

The young people’s reports seem to describe the therapists’ attempts to make transference interpretations, using the therapy relationship as a tool to discuss unconscious thoughts. These interventions, however, seemed to not resonate with the young people at given moments in the therapy process.

The struggles related to therapy interventions were not limited to the ones focusing on the dyads’ relationships. Daniel, for example, stated:

**Daniel:** One time she asked me what I was doing, like what I had done that day and I said I was on the computer for about half an hour, and then she asked me what I was doing on the computer, and I said I was playing a game. And then she asked me to describe the game and I described it and she started making analogies for other things I said about the game, and I said ‘no, I just played it for half an hour, it’s not my entire life’.

From this data extract, this young person illustrates how his therapist would attribute symbolic meanings to some experiences he did not see as having such. In different interviews, those types of intervention were employed concerning diverse types of content, such as dreams and media content.

In addition, the young people also described some emotional reactions when facing comments from their therapists that were deemed inaccurate:

**Daniel:** Sometimes I get frustrated because she will say things … – she’ll come up with a theory for why I’m thinking this or saying this and that will just not be right. And then I’ll try and say that, but it sometimes doesn’t sink in. And sometimes things are looked into too in-depth like I find it frustrating that I mentioned something in passing and then that is explored, y’know, as if it’s affecting me. Like I mention that I saw something … in the news and then that’ll be picked apart when I don’t really see there’s any point in that.

According to Daniel, his therapist’s interventions at times would make him feel frustrated, as they would deviate the therapy’s focus from the topics he considered more important to be discussed in the hour. Another type of reaction is presented by Anna:

**Anna:** She [was] always saying… I remember how even at the end how if I'm gonna think… if she still thinks about me or when I went [home] for Christmas so I didn’t see her for two weeks she … asked me if I'm gonna be … over these 2 weeks thinking if she thinks about me or if she remembers me … and I always thinking… I never thought about that, so it was kind of… weird for me for her to ask things like that.

This young person’s comments seem to describe a degree of confusion or awkwardness following some of her therapist’s inferences about her own thoughts.

In general, from the young people’s perspectives, some comments from their therapists would not make sense to them, such as establishing connections between the therapy relationship and relationships outside therapy and attributing symbolic meaning to everyday activities or dreams. Furthermore, they also reported that these interventions would come across as imprecise at times, and such imprecisions lead to feelings of frustration or confusion.

## 4 Discussion

The present study aimed to investigate how young people with major depressive disorder who remained clinically depressed after short-term psychoanalytic psychotherapy, their therapists, and parents made sense of the experience of psychotherapy. By analyzing semi-structured interviews using a descriptive-interpretative approach, three main themes emerged. The different themes evidenced positive aspects of the therapy process according to the different participants, as well as their own understanding of how helpful therapy potentially was and some setbacks and struggles with aspects of the therapeutic interventions.

The first theme, “Therapy as a safe space,” evidenced that young people, their therapists, and parents appreciated therapy as a space where the patients could express their thoughts and feelings that they would not be able to in other contexts. This theme was surprisingly present in our sample, considering that our study addressed cases where young people remained clinically depressed after follow-up. In general, this theme suggests that “unsuccessful” therapy does not reflect a negative experience in psychotherapy, just like “successful” therapies do not necessarily reflect positive experiences (de Smet et al., 2021).

Our findings are to some extent similar to the ones found by McElvaney and Timulak (2013). By studying the perspectives of 11 adult patients who received a treatment combining Cognitive-behavioral Therapy and Person-centered approaches, these authors found that even in poor outcome cases the patients were found to have positive experiences of therapy. According to their findings, poor outcome cases specifically appreciated therapy as a tool to raise awareness of problematic functioning and mastering problematic experiences. Furthermore, these patients also valued the guidance provided by their therapists’. While we also found positive experiences among our cases, with participants referring to therapy as a “safe space,” this was more related to issues of self-expression (including how young people could and should behave in different environments) and trust (e.g., non-judgmental stance and confidentiality). Taken altogether, our results indicate that positive experiences of psychotherapy can also be seen in the treatment of young people and that experiencing therapy as a “safe space” by itself may not reflect a reduction in the patients’ symptoms.

In theme 2, “Can short-term psychotherapy ever be enough?”, the participants presented their perspectives on the curative potential of STPP. While parents and therapists tended to be more positive concerning the outcomes achieved after STPP, the young people’s perspective was more reserved. We identified in the adolescents’ interviews either a degree of fatalism or an understanding of the limitations of the approach offered. According to some adolescents, their depression and overall problems were too overwhelming in their lives in comparison to the weekly hour offered in the treatment program. In addition, some young people reported believing that therapy was only helpful while it lasted, only allowing for temporary improvement.

On one hand, these young people’s perspectives might point to a “real” need for more intensive psychotherapy. In a systematic review and meta-regression on the treatment of adult depression, Cuijpers et al. (2013) found that the association between treatment overall length and outcomes was negligible but having more frequent sessions per week (two weekly sessions versus one weekly session) seemed to increase the likelihood of positive outcomes. On the other hand, the participants’ fatalistic accounts might also indicate personal characteristics that could be associated with more severe depressive symptoms. Fatalism (that is, the belief that events are set to happen regardless of actions) seems to be significantly associated with depressive symptoms (Shahid et al., 2020), which could, in turn, impact therapeutic progress.

The discrepancies in the participants’ reports could be understood considering outcome studies including different stakeholders. When rating young people’s internalizing symptoms, young people seem to provide higher scores about their own difficulties when compared to their parents (Orchard et al., 2017, 2019; Makol and Polo, 2018; Serafimova et al., 2021). However, it is worth noting that parents and therapists accounted for their perception of change based on other potentially meaningful outcomes, such as academic and social functioning and coping skills (Krause et al., 2020). Hence, these cases also indicate that the understanding of “poor outcome” in psychotherapy is more nuanced than a simple “failure.”

The third and last theme, named “Therapists making links and connections that did not make sense to the young people,” was only raised by young people and described moments in the process where the patients would not understand the reasons for some given interventions, or even consider them as inaccurate or confusing.

On one hand, these reports seem to describe therapists who were employing saturated (i.e., explicitly transferential, or more “direct”) interpretations when treating these young people (Ferro, 2006). Considering that adolescence is a developmental stage characterized by a specific process of separation-individuation (Adatto, 1966). Therefore, young people might present resistance over those interventions, considering their regressive nature, or find them triggering (Swift and Wonderlich, 1990; Laufer, 1997). For example, Della Rosa and Midgley (2017) examined transference interpretations concerning the end of therapy among depressed adolescents in the IMPACT study STPP arm. These authors found two types of responses elicited when therapists directly linked the adolescents’ life events or relationships with therapy: adolescents showed either a degree of dramatization—describing over-pessimistic or catastrophic expectations for their lives after therapy ended—or down-playing—stating that they feel fine about the treatments’ ending and that their problems have already been solved. In that context, direct transference interpretations could induce anxiety and self-consciousness in adolescents, hindering their capacity for in-depth self-reflection and effective understanding (Briggs, 2019).

Along similar lines, another possible interpretation concerning this theme is that those therapists were—at least at moments—not adopting a mentalizing (or “not-knowing”) stance (Bateman and Fonagy, 2006). In that regard, the young people’s reports seemed to describe interactions where their therapists jumped to conclusions, putting themselves in a position where they knew more about the patients’ minds than the patients themselves. In that sense, although the interventions employed seemed to be aligned with the STPP manual (Cregeen et al., 2017), they were not always received by the young people as intended. Regarding this issue, a meta-analysis on the relationship between treatment adherence and outcomes in child and adolescent psychotherapy found that adherence only accounted for a small effect size, suggesting that applying prescribed therapy practices plays a minor role in therapy success (Collyer et al., 2020). Overall, this indicates that therapists’ flexibility to their patients’ specific needs might be key to effective treatments, instead of rigid loyalty to a given treatment protocol.

It is worth observing that both themes 2 and 3 encompassed characteristics described by O’Keeffe et al. (2019) as part of a “dissatisfied” drop-out. In that study, the authors examined the perspectives of depressed young people who dropped out from the short-term psychotherapies within the IMPACT trial. Some patients in the “dissatisfied” group reported that they dropped out because they felt they were not benefitting from therapy (like Marcus and Riley in Theme 2), and some within the STPP arm stated that some of the therapists’ interpretations did not make sense to them (Like Marcus, Anna, and Daniel in Theme 3). In the present sample, these characteristics did not make the patients interrupt their treatments, since all were treatment completers. One potential hypothesis on why these patients stayed in treatment is that even though some of them did not think they were benefitting from therapy *per se*, they appreciated the sense of safe space it fostered, as present in Theme 1. Furthermore, even though some young people reported finding some interventions pointless or inaccurate, it could mean that they were not overwhelming characteristics of their treatments, but rather facets of a broader experience.

### 4.1 Strengths and limitations

The present study has a series of strengths and limitations. Firstly, by drawing its data from a randomized trial, counting with standardized research protocols, the participants had a fairly homogeneous experience: all treatments took place in London CAMHS, following the same treatment manual, and the qualitative interviews followed a similar structure across participants.

Nevertheless, we highlight that there are also some limitations in terms of the conclusions we can draw from this theme considering our dataset. While patients and parents were interviewed by the end of therapy (week 36) and 1 year after the treatment ended (week 86), the therapists’ interviews took place only on week 36. Hence, therapists did not have contact with patients and therefore did not have evidence to know how the young people were presenting themselves 1 year after therapy ended. Perhaps having longer-term contact with the patients could have led therapists to have a different understanding of how they changed—or not—following the intervention. Likewise, we did not have any data concerning the young people after week 86. Therefore, we do not know anything about the cases’ progression after 1 year from the end of treatment. Also due to the nature of the database available, we did not have any information concerning the therapists (e.g., age, gender, and years of clinical experience).

It is worth noting that this study was part of a larger investigation, which also analyzed the same cases’ psychotherapy process. For this purpose, we selected cases according to data availability, considering the availability of session recordings and qualitative interviews. By selecting patients who had more recordings and who had participated in more interviews, we might have indirectly selected young people and families who were more compliant and who had more positive views regarding the research protocol and their own treatment. Examining the same research questions with adolescents who dropped out or with participants who had a more dissatisfied or conflicted relationship with their therapists and the research program could also be valuable in understanding other facets of therapy “failure.” Furthermore, this study broadly addressed participants who remained clinically depressed after STPP, with some patients even showing some limited degree of improvement in their clinical symptoms. Investigations addressing young people who had their symptoms worsened after psychological treatments could also shed light on other experiences of psychotherapy.

Lastly, this investigation only included the perspectives of the parents of two young people. Despite the IMPACT and IMPACT-ME being large-scale studies within the field of youth psychotherapy, only a small percentage of cases was eligible to participate in this particular investigation, leading to limited data availability (just a third of participants were randomized into STPP, IMPACT-ME interviews were only conducted in one of the locations from the main trial, the number of participants who had “poor” outcomes was smaller than the ones who achieved “good” outcomes, and perhaps participants in cases with suboptimal outcomes were less likely to participate in the IMPACT-ME interviews). Further studies addressing parental perspectives on youth psychotherapy can be valuable in widening our understanding of how they experience the therapy process.

### 4.2 Clinical implications

From our findings, we draw some clinical implications. Firstly, therapists should be mindful that patients’ positive experiences of therapy do not necessarily reflect effective therapy. In that sense, when keeping track of a given treatment, one should include paying attention to multiple indicators that go beyond the therapy relationship and the patient’s symptoms.

Secondly, young people’s perspectives on their outcomes may differ from their therapists’ and parents’. Giving voice to the patients’ perspectives on their progress (or lack of it) can be useful in determining potential areas that need attention (e.g., symptoms that were not perceived by parents or therapists, and not brought up spontaneously during therapy).

Lastly, we highlight that the use of some direct transference interpretations may elicit negative reactions in depressed adolescents, including feelings of confusion and inadequacy. Employing “unsaturated”—or tentative—interpretations would be favored in key moments, since they open the way to new understandings that are mutually built between the dyad, rather than being narrow, limiting, and perhaps even intimidating. In this approach, talking about the patient’s issues in a more open and general way could be more effective than directly connecting them to the therapy relationship.

## Data Availability

All data is held by Anna Freud following the GDPR principles. Requests to access these datasets should be directed to NM, nick.midgley@annafreud.org.
